# Case–control, kin-cohort and meta-analyses provide no support for *STK15* F31I as a low penetrance colorectal cancer allele

**DOI:** 10.1038/sj.bjc.6603382

**Published:** 2006-09-26

**Authors:** E L Webb, M F Rudd, R S Houlston

**Affiliations:** 1 Section of Cancer Genetics, Institute of Cancer Research, Surrey, UK

**Keywords:** *STK15*, polymorphism, colorectal cancer, risk

## Abstract

Recently, homozygosity for T91A single-nucleotide polymorphism (SNP) in the serine/threonine kinase *(STK15)* gene, which generates the substitution F31I has been proposed to increase the risk of a number of tumours including colorectal cancer (CRC). To further evaluate the relationship between *STK15* F31I and risk of CRC, we genotyped 2558 CRC cases and 2680 controls for this polymorphism. We found no evidence that homozygosity for the *STK15* 31I genotype confers an increased risk of CRC (odds ratio=0.95, 95% confidence interval (CI): 0.74–1.24). We also conducted a kin-cohort analysis to assess risk among first-degree relatives of the CRC cases. The hazard ratio for I/I homozygotes compared to F/F homozygotes was 1.65 (95% CI: 0.39–3.17). A meta-analysis of our case–control data and three previous studies also provided no evidence of an elevated risk of CRC associated with homozygosity. These data provide no support for the hypothesis that sequence variation in *STK15* defined by SNP F31I *per se* confers an elevated risk of CRC.

Serine/threonine kinase (*STK15*; MIM:603072) is a regulator of mitotic chromosomal segregation (Dutertre *et al*, 2002) and plays a role in the development of malignancy (Bischoff *et al*, 1998; Zhou *et al*, 1998; Ewart-Toland *et al*, 2003). Recently, the T91A single-nucleotide polymorphism (SNP) in *STK15*, which generates the substitution F31I, and has been reported to influence genomic instability (Ewart-Toland *et al*, 2003; Kimura *et al*, 2005), has been proposed as a low penetrance variant for a number of tumours including colorectal cancer (CRC) (Ewart-Toland *et al*, 2003, 2005). To further evaluate the relationship between *STK15* F31I and risk of CRC, we genotyped 2558 CRC cases and 2680 controls for this polymorphism. We also conducted a kin-cohort analysis to assess risk among first-degree relatives of the CRC cases.

## MATERIALS AND METHODS

Unselected CRC cases with histologically confirmed colorectal adenocarcinomas (1471 men; 1087 women; mean age at diagnosis 61 years; standard deviation (s.d.)±11.4) were ascertained through over 100 centres in the UK. Detailed family histories were obtained from cases by previously validated questionnaire. Controls (836 men; 1844 women; mean age 59 years; s.d.±10.9) were the spouses or friends of patients with malignancies ascertained as part of ongoing National Cancer Research Network genetic epidemiological studies (1999–2004; *n*=1067), the Royal Marsden Hospital Trust/Institute of Cancer Research Family History and DNA Registry (1999–2004; *n*=1021) and UK Study of Breast Cancer Genetics (1999–2004; *n*=592) all established within the UK. None of the controls had a personal history of malignancy at time of ascertainment. As we are not seeking to evaluate environmental interactive effects, the theoretical possibility of ‘overmatching’ on environmental factors by using spouse and friend controls is unlikely to impact on study findings. All cases and controls were British Caucasians, and there were no obvious differences in the demography of cases and controls in terms of place of residence within the UK. Blood samples were obtained with informed consent and ethical review board approval in accordance with the tenets of the Declaration of Helsinki.

Analysis of *STK15* F31I was undertaken as part of our ongoing analyses to identify low penetrance alleles based on the National Study of Colorectal Cancer Genetics Trial (2004). DNA was extracted from blood samples using conventional methodologies and quantified using PicoGreen (Invitrogen, Carlsbad, CA, USA). Genotyping was performed using the Illumina Sentrix Bead Array system according to the manufacturer's protocols. Further information on the assays is available on request.

Risks were estimated by odds ratios (ORs) using unconditional logistic regression, and associated 95% confidence intervals (CIs) computed. Meta-analysis of this and other published studies was conducted using standard methods for combining estimates of ORs based on the weighted sum of the log estimates with the inverse of the variance of the estimate of the weight. Cochran's *Q* statistic (Cochran, 1954) to test for heterogeneity and the *I*^2^ statistic (Higgins *et al*, 2003) to quantify the proportion of the total variation owing to heterogeneity were calculated. To incorporate within-study and between-study variability, we used DerSimioian and Laird's method (DerSimonian and Laird, 1986) for calculating random effects summary ORs and their associated 95% CIs. All computations were undertaken in STATA (version 8.2, Stata Corporation, College Station, TX, USA).

We also performed a kin-cohort analysis to compare the CRC risk in first-degree relatives of *STK15* I homozygotes with that in first-degree relatives of F homozygotes. Proband genotypes and the SNP allele frequency were used to infer genotypes of relatives. Age-specific cumulative CRC distributions in first-degree relatives were estimated using a marginal likelihood approach (Chatterjee and Wacholder, 2001), implemented in MatLab version 7.0.1 (MathWorks, Natick, MA, USA). Bootstrap estimates for the hazard ratio (HR) were then computed, using 1000 resamples of the data, and used to generate a 95% CI.

## RESULTS

There was no evidence of population stratification as the genotype distribution of F31I in controls satisfied Hardy–Weinberg equilibrium (*P*=0.63). We found no evidence that homozygosity for the *STK15* I genotype confers an increased risk of CRC (cases: F/F=1564, F/I=880 and I/I=114; controls: F/F=1667, F/I=888, I/I=125; OR=0.95, 95% CI: 0.74–1.24). As there is no *a priori* reason to assume a recessive model of CRC susceptibility, we also computed the risk associated with heterozygotes using the F/F genotype as the reference group. The OR under this model was 1.06 (95% CI: 0.94–1.19).

**Figure 1:**
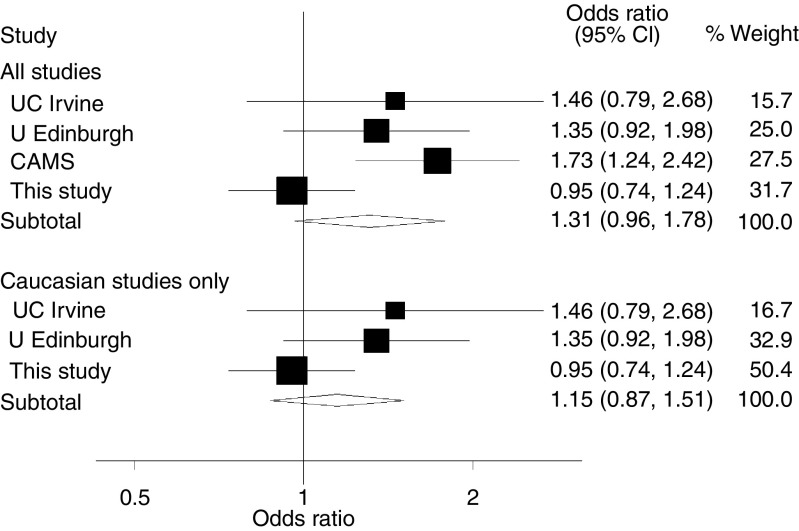
Forest plot of the risk of CRC associated with STK15-I/I homozygosity from comparison of I/I *vs* I/F and F/F genotypes combined for (**A**) all studies and (**B**) all Caucasian studies. Shaded squares denote ORs, with their size being proportional to the inverse variance of the study-specific estimate. Horizontal lines represent 95% CIs. The diamond denotes the combined random effects estimate and its associated 95% CI. The vertical line indicates the null effect (OR=1.0).

Three other studies have compared the frequency of *STK15* F31I genotypes in cases and controls (Ewart-Toland *et al*, 2005); two based on predominantly Caucasians and one based on Han Chinese. Figure 1 shows estimates of the recessive homozygote risk from this study and from published studies. The study based on Han Chinese was alone in showing a significant positive association and was the only study where results differed significantly from our analysis. The pooled OR estimate was 1.31 (95% CI: 0.96–1.78); interpretation of this is not, however, clear as the estimates in different studies are clearly heterogeneous (Cochran's *Q*=8.21, *P*=0.042, *I*^*2*^=64%). Restricting the meta-analysis to the three studies based on Caucasians, between-study heterogeneity was less evident (Cochran's *Q*=3.1, *P*=0.21, *I*^*2*^=35%). The pooled estimate of the OR was 1.15 (95% CI: 0.87–1.51).

The 2558 cases reported a total of 14 649 first-degree relatives of whom 445 (3.0%) had been diagnosed with CRC. The HR for I/I homozygotes compared to F/F homozygotes was 1.65 (95% CI: 0.39–3.17).

## DISCUSSION

It has previously been proposed that the *STK15* F31I polymorphism confers a 1.5-fold increase in risk of CRC (Ewart-Toland *et al*, 2005). Even though our study had over 90% power to detect this magnitude of increased risk at significance level 5%, we found no evidence to support the hypothesis that F31I is a CRC susceptibility allele. Moreover, the meta-analysis we conducted of 9309 subjects also provides no evidence of an increased risk. There was, however, a high degree of variation in the ORs estimated by individual studies. The frequency of the I-allele is markedly different in Caucasians and Han Chinese (21 and 61% respectively), thus it may be inappropriate to regard the different ethnic groups as genetically similar. As the frequency of genotypes in controls in the other studies are not significantly different from those expected, there is little evidence that overt population stratification underpins study heterogeneity. Although there is some data to suggest that F31I has functional cellular consequences (Ewart-Toland *et al*, 2003; Kimura *et al*, 2005), direct causality has not been demonstrated. It is possible that population-specific linkage disequilibrium with another functional variant could be operating. Some support for this is provided by the observation of Lo *et al* (2005) that in the Taiwanese population the risk of breast cancer associated with F31I is mediated through an extended haplotype. Finally, as with all association studies a simple but unattractive explanation for the observed heterogeneity is publication bias. In summary, collectively these data provide no support for the hypothesis that sequence variation in *STK15* defined by the nonsynonymous SNP F31I *per se* confers an elevated risk of CRC.

## References

[bib1] Bischoff JR, Anderson L, Zhu Y, Mossie K, Ng L, Souza B, Schryver B, Flanagan P, Clairvoyant F, Ginther C, Chan CS, Novotny M, Slamon DJ, Plowman GD (1998) A homologue of *Drosophila* aurora kinase is oncogenic and amplified in human colorectal cancers. EMBO J 17: 3052–3065 9606188 10.1093/emboj/17.11.3052 PMC1170645

[bib2] Chatterjee N, Wacholder S (2001) A marginal likelihood approach for estimating penetrance from kin-cohort designs. Biometrics 57: 245–252 11252606 10.1111/j.0006-341x.2001.00245.x

[bib3] Cochran W (1954) The contribution of estimates from different experiments. Biometrics 10: 101–129

[bib4] DerSimonian R, Laird N (1986) Meta-analysis in clinical trials. Control Clin Trials 7: 177–188 3802833 10.1016/0197-2456(86)90046-2

[bib5] Dutertre S, Descamps S, Prigent C (2002) On the role of aurora-A in centrosome function. Oncogene 21: 6175–6183 12214247 10.1038/sj.onc.1205775

[bib6] Ewart-Toland A, Briassouli P, de Koning JP, Mao JH, Yuan J, Chan F, MacCarthy-Morrogh L, Ponder BA, Nagase H, Burn J, Ball S, Almeida M, Linardopoulos S, Balmain A (2003) Identification of Stk6/STK15 as a candidate low-penetrance tumor-susceptibility gene in mouse and human. Nat Genet 34: 403–412 12881723 10.1038/ng1220

[bib7] Ewart-Toland A, Dai Q, Gao YT, Nagase H, Dunlop MG, Farrington SM, Barnetson RA, Anton-Culver H, Peel D, Ziogas A, Lin D, Miao X, Sun T, Ostrander EA, Stanford JL, Langlois M, Chan JM, Yuan J, Harris CC, Bowman ED, Clayman GL, Lippman SM, Lee JJ, Zheng W, Balmain A (2005) Aurora-A/STK15 T+91A is a general low penetrance cancer susceptibility gene: a meta-analysis of multiple cancer types. Carcinogenesis 26: 1368–1373 15802297 10.1093/carcin/bgi085

[bib8] Higgins JP, Thompson SG, Deeks JJ, Altman DG (2003) Measuring inconsistency in meta-analyses. BMJ 327: 557–560 12958120 10.1136/bmj.327.7414.557 PMC192859

[bib9] Kimura MT, Mori T, Conroy J, Nowak NJ, Satomi S, Tamai K, Nagase H (2005) Two functional coding single nucleotide polymorphisms in STK15 (Aurora-A) coordinately increase esophageal cancer risk. Cancer Res 65: 3548–3554 15867347 10.1158/0008-5472.CAN-04-2149 PMC2536656

[bib10] Lo YL, Yu JC, Chen ST, Yang HC, Fann CS, Mau YC, Shen CY (2005) Breast cancer risk associated with genotypic polymorphism of the mitosis-regulating gene Aurora-A/STK15/BTAK. Int J Cancer 115: 276–283 15688402 10.1002/ijc.20855

[bib11] Zhou H, Kuang J, Zhong L, Kuo WL, Gray JW, Sahin A, Brinkley BR, Sen S (1998) Tumour amplified kinase STK15/BTAK induces centrosome amplification, aneuploidy and transformation. Nat Genet 20: 189–193 9771714 10.1038/2496

